# Open innovation and confidentiality agreements as key factors of innovative performance in the manufacturing and service industries

**DOI:** 10.1371/journal.pone.0303802

**Published:** 2024-05-20

**Authors:** Fernando Barrios Aguirre, Diana Maritza Alvarez Ovalle, Nancy Milena Riveros Chávez, Carla Johana Martinez Garcia

**Affiliations:** 1 Escuela de Negocios, Fundación Universitaria Konrad Lorenz, Bogotá, Colombia; 2 School of Management, Business and Economics Sciences, Fundación Universitaria del Área Andina, Bogotá, Colombia; 3 School of Management, Business and Economics Sciences, Fundación Universitaria del Área Andina, Pereira, Colombia; University of Calabria Department of Mechanical Engineering Energy Engineering and Management Engineering: Universita della Calabria Dipartimento di Ingegneria Meccanica Energetica e Gestionale, ITALY

## Abstract

The innovative performance of manufacturing and service companies can be impacted by the existing relationship between open innovation (OI) and the generation of confidentiality agreements (NDAs) as a tool for the protection of intellectual property. Based on the analysis of a cross-sectional sample of 6,798 industrial companies (2019–2020) and 9,304 companies in the service sector (2017–2019) that are part of the directory of the National Administrative Department of Statistics (DANE) in its Technological Innovation and Development Survey (EDIT and EDITS), it can be suggested that the interaction of these two variables (OI and NDAs) generate positive effects for the manufacturing industry but negative ones for the service sector. It could be deduced that the positive effect is due to the greater tradition of OI in the manufacturing industry and the negative effect to the caution that the service sector presents when collaborating with external actors.

## Introduction

Open Innovation (OI) is a collaborative approach to innovation that allows working with external partners to create shared value [1]. This OI approach is different from the traditional closed innovation approach, which focuses on in-house R&D and technology patents for exclusive use. A clear example of open innovation (IO) in the manufacturing industry is the association with suppliers that allows the development of new technologies and/or materials. Another example is the association with universities to investigate new manufacturing processes. In the case of the service industry, companies can work in collaboration with startups and develop new digital platforms or with their clients to co-create new service offerings.

OI has become an inevitable strategy in terms of business competitiveness, different authors examine the concept and types of open innovation practices, as a fundamental axis of manufacturing and service companies [2,3], however, it is necessary to understand that there are different OI practices including inbound, outbound and coupled innovation [4] since by combining these with corporate risk and organizational strategy, a competitive advantage can be generated [5]. Open innovation can take many forms, including co-creation with the customer, informal networking, college scholarships, crowdsourcing, joint ventures, market-ready product sales, donations to commons, and spin-offs [6].

The OI produces different commercial benefits, as indicated by some research, with access to knowledge and experience being the two fundamental axes of development and management that allow the reduction of research and development costs, improving the time to market [1,7]. OI allows companies to tap into a broader pool of knowledge and experience than they would otherwise have access to through in-house research and development alone, this is one of the main benefits of OI. With this in mind, companies can take advantage of the experience, resources, and capabilities of their partners to accelerate innovation, reduce costs, and improve the quality of their products and services by working with external partners.

This protection of confidential and sensitive data and intellectual property rights, constitutionally protected, creates the ideal conditions for OI, since business relationships are marked by objective good faith that guides the behavior of the parties throughout the contractual iter and ranges from the voluntary acceptance of the contractual stipulations until after its termination, within the framework of "loyalty, diligence, honesty, probity, transparency" [8], creating the obligation to abide by contractual clauses such as the *rebus sic stantibus*, the prohibition of obtaining harm from others, and the protection of the contractual *synallagma* among other private provisions under private law.

The innovative performance of manufacturing and service companies can be impacted by the existing relationship between open innovation (OI) and the generation of confidentiality agreements (NDAs) as a tool for the protection of intellectual property. Based on the analysis of a cross-sectional sample of 6,798 industrial companies (2019–2020) and 9,304 companies in the service sector (2017–2019) that are part of the directory of the National Administrative Department of Statistics (DANE), it can be suggested that the interaction of These two variables (OI and NDAs) generate positive effects for the manufacturing industry but negative ones for the service sector. It could be deduced that the positive effect is due to the greater tradition of OI in the manufacturing industry and the negative effect to the caution that the service sector presents when collaborating with external actors.

This article is divided into four sections. The first section presents the review of the literature that inspires this research and focuses on showing how open innovation and confidentiality agreements are related to innovative performance in the manufacturing and service industries. The second section has the econometric model and data and discusses the strategy for identifying the estimated effects in the zero-inflated Poisson model. The third section shows the descriptive statistics and presents and analyzes the research results. Finally, the fourth one presents the conclusion.

## Literature review

Open innovation (OI) is a collaboration that can be included in the research and development phase, co-creation of products and services, and joint exploration of new markets. Open innovation can be very effective in expanding the scope of innovation, drawing on the knowledge and experience of a wide variety of actors and it can enable to obtain competitive advantage and take advantage of market opportunities [3].

OI has become an increasingly popular approach for driving innovation and improving competitiveness in the manufacturing and service industries. However, the collaborative nature of open innovation can create challenges around confidentiality and intellectual property. Open innovation (OI) is a paradigm that suggests that firms can find useful ideas not only inside but outside of them [1]. For this reason, companies collaborate with external partners, such as customers, suppliers, universities, innovation and research center, business incubators, spin off, and startups, to generate valuable ideas and innovative solutions, it implies the collaboration between interdependent actors where the trust in capabilities of others determine the value creation [9].

The OI can be very effective in expanding the scope of innovation, based on the knowledge and experience of a wide variety of actors that allows obtaining a competitive advantage and taking advantage of market opportunities [3], developing new products and new processes and creating value, which can lead to more focused and effective innovation [10] which makes OI long-term strategy implemented by the firms as a result of organizational learning that generates sustainable competitive advantages and increases in a knowledge management capacity [2].

Consequently, it is strategic for firms that their trade secrets and innovations are protected under the contractual protection of instruments for the protection of intellectual property (PPI). Protecting intellectual property is essential for companies to maintain their competitive advantage and their ability to innovate in the future, especially since OI develops an ambiguous and complex scenario in social terms; consequently, it becomes a generator of imitation barriers for competitors and a driver for the protection of "informal" intellectual property. This informal approach is especially attractive to companies in developing economies, where formal institutional protection of the intellectual property is weak [11].

In this sense, the firms implement legal figures such as confidentiality contracts that solve the demanding challenges that firms face when there are several actors involved in the development of innovation [12]. Talking about them means establishing a relationship of trust between the company and its external partners, allowing them to share sensitive information without fear of it being disclosed to third parties without consent, this being a solid driver for OI [11,12]; in the understanding that trust improves the exchange and acquisition of resources while reducing conflicts [13,14].

In other words, OI and confidentiality agreements promote the innovative performance of companies by allowing them to collaborate with a broader set of external actors and protect their innovations, in contrast, not implementing PPI strategies could trigger an adverse effect on the OI and innovative performance [15]. For this reason, companies must be willing to share their knowledge and thus collaborate with other interested parties to ensure the appropriability of innovation in a safe manner, which is known as formal OI [10].

In terms of confidentiality agreements, the literature recognizes the importance of NDAs due to the fact that knowledge is a critical resource for the innovation, competitiveness and survival of organizations [16], and their constitutional support and their legal implications, as well as the potential benefits and drawbacks of using such agreements [17,18].

In the case of Colombia, confidentiality agreements are based on the principle of good faith. For Chinchilla [8], "good faith is a" higher principle of law ", and therefore constitutes one of the founding elements of our legal tradition". This constitutional principle is closely related to the principles of law, legitimate expectations, and legal certainty. This principle of natural law is enshrined in the Colombian Constitution in its article 83, this principle privileges "loyalty, rectitude, balance, honesty, diligence, transparency, protection of trust" in contractual relationships establishing a legal bond of legitimate trust, which reaffirms that legitimate trust derives from the principle of good faith, as the constitutional court has repeatedly indicated in judgments C-478/1998, T-020/2000 and T-1094/2005 [19].

On the other hand, confidentiality agreements are essential to protect a company’s trade secrets and innovations. These agreements establish a relationship of trust between the company and its external partners, allowing them to share sensitive information without fear of it being disclosed to third parties without consent [10]. Protecting intellectual property is critical for companies to maintain their competitive advantage and ability to innovate well into the future, above all because OI presses causal ambiguity and social complexity, creates imitation barriers for competitors, and provides "informal" intellectual property protection. This informal approach is especially attractive to companies in developing economies, where formal institutional protection of the intellectual property is weak [11].

But there is a negative side, since the contractual effort could be an obstacle in the medium term to generate new OI processes because the confidentiality of this can reduce the levels of transparency, especially regarding the use of previously obtained results becoming a barrier to the promotion of new OI projects [20], in fact, authors such as Hallberg and Brattström [21], Noh and Lee [22] and Toma et al. [23] agree that intellectual property makes it difficult to share knowledge.

In summary, open innovation and confidentiality agreements are two critical factors in driving innovative performance in the manufacturing and service industries. Companies must strike a balance between collaborating with external partners and protecting their confidential information and trade secrets to fully reap the benefits of open innovation. By doing so, they can accelerate innovation, reduce costs, and improve the quality of their products and services, which ultimately leads to greater competitiveness and profitability.

### Combine both

Together, open innovation and non-disclosure agreements can boost companies’ innovative performance by enabling them to collaborate with a broader set of stakeholders and protect their innovations in the process. Additionally, open innovation can help companies identify new business opportunities and improve their understanding of the market, which can lead to more focused and effective innovation. Overall, these strategies are critical for companies to maintain their competitive edge and continue to innovate in today’s ever-evolving marketplace. Despite this, there is a negative side, since the contractual effort could be an obstacle to generating other OI processes because the confidentiality of the agreement decreases the transparency to obtaining information and starting a new OI project using the results of the previous ones [20].

Innovation is a critical factor in driving competitiveness and profitability in the manufacturing and service industries. To stay ahead of the competition, companies must continually develop new products, services, and processes. Open innovation, a collaborative approach that involves working with external partners to co-create value, has become an increasingly popular approach for driving innovation and improving competitiveness. However, open innovation can create challenges around confidentiality and intellectual property, as companies must share their knowledge and expertise with external partners. Discussion is current, for example some authors study some way different relation, and argue that there exists a need of protecting intellectual property and this is a boost of open innovation [11].

In today’s rapidly changing business environment, innovation is key to success and survival in both the manufacturing and service industries. Companies are increasingly turning to open innovation, a collaborative approach that involves working with external partners such as customers, suppliers, universities, and startups, to drive innovation and improve their competitive position [24]. Open innovation can take many forms, including partnerships, joint ventures, licensing agreements, and crowdsourcing. However, open innovation can also create challenges around confidentiality and intellectual property, which must be managed through robust confidentiality agreements.

Companies must balance the benefits of collaboration with the need to protect their confidential information and trade secrets. In this article, we will explore how open innovation and confidentiality agreements are key factors in driving innovative performance in the manufacturing and service industries.

Faced with the possible reasons that imply that a confidentiality agreement facilitates innovative performance, there is the constitutional and protective development that has been generated around private law, since in commercial law contracts constitute the law for the parties covered by the principles of good faith, the principle of legitimate trust and the legal certainty that arises from the contractual relationship. Explaining the foregoing, it should be noted that in Colombia the principle of good faith is constitutionalized in Article 83 of the 1991 political constitution. The principle of Segura legitimate trust indicates that this principle derives from the principle of good faith taking into account jurisprudence and doctrine, for which it cites judgments C-478/1998, T-020/2000, and T-1094/2005 of the Constitutional Court [19].

In sum, open innovation and confidentiality agreements are two critical factors in driving innovative performance in the manufacturing and service industries. Companies must strike a balance between collaborating with external partners and protecting their confidential information and trade secrets to fully realize the benefits of open innovation. By doing so, they can accelerate innovation, reduce costs, and improve the quality of their products and services, ultimately leading to improved competitiveness and profitability.

## Method, model, identification strategy and data

There are many observations of zero in the total innovations (76.3% manufacturing and 77.8% services), that is, not all companies innovate despite the benefits of doing so. However, having many non-innovative signatures is not a problem if the fact that the variable takes the value 0 can be interpreted in two different ways, in such a way that we maintain the condition that occurs for a certain data generation process. When this is the case, zero-inflated models (Zero-inflated Poisson ZIP, model or Zero-inflated negative binomial ZINB) can provide better results than Poisson and/or negative binomial models, since they do not take into account in the estimation of these possible differentiating aspects, while the inflated zeros assume that the dependent variable is the product of a binary law and a Poisson law or negative binomial [25].

### Data

The sample is made up of the number of companies reported in the Technological Development and Innovation Survey for the manufacturing and services industry (EDIT and EDITS). This database is made up of a cross-section of 6798 industrial companies (2019–2020) and 9,304 companies in the services sector (2017–2019), which are part of the DANE directory. The objective of these surveys is to characterize the dynamics of technological development of the manufacturing and service companies in Colombia, in terms of intensity and trajectory of innovation and technological development activities, to evaluate the incidence of public policy instruments, and to establish the types of occupational profiles applied in the different areas or departments of the companies.

In this study, innovative performance in products, processes, markets, and organizations is used as a dependent variable, in a binary context (1 = yes, it innovates; 0 = otherwise) and on a discrete, non-negative scale (innovation count). Counting marketing innovations, this variable is characterized by a high number of zero observations and few observations with high positive values, so it could be inferred that it follows a negative Poisson or binomial distribution. From the dependent variable, it will be observed that there is a causal relationship between the conglomerates and the variation of innovative performance.

From the information available in the EDIT and EDITS, the dependent and independent variables to use are the following:

Dependent variables:

1. Count of innovations in products, processes, markets, and organizations of the firms.
2. Binary of not innovating in products, processes, markets, and organizational firms (1 = does not innovate; 0 = Innovates).

Independent variables. To explore:

1. Company size: Number of company employees in logarithms.
2. Source of vertical ideas: Equal to 1 if the company uses customers or suppliers as sources of information for innovation. Equal to 0 otherwise.
3. Source of ideas from universities and research centers: Equal to 1 if the company uses universities and R&D centers (Technological Development Centers -CDT and Research Centers) as sources of information for innovation. Equal to 0 otherwise.
4. Demand Push: It is a binary variable, equal to one if the company expresses as very important the improvement in the quality of the goods or services and the expansion in the range of goods or services offered [26]. Equal to 0 otherwise.
5. Highly qualified personnel refers to employed personnel with master’s and doctoral degrees over the total personnel.
6. Qualified personnel: Refers to employed personnel with undergraduate training and specialization over the total personnel.
7. R&D expenses: Logarithm of the investment in internal and external R&D activities.
8. Confidentiality Agreements: A dummy variable related to the existence of Confidentiality Agreements in the industry and services sector.
9. Open Innovations: A dummy variable that includes the resource of any of these sources of information for science, technology, and innovation activities: Clients, Suppliers, technological development centers, autonomous research centers, incubators for technology-based companies, and Training centers and/or technoparks, regional productivity centers, and universities.

## Results

Tables 1 and 2 present the descriptive statistics of the main variables of the information obtained in the survey carried out on 6798 Colombian manufacturing firms and 9304 firms in the services sector. According to the descriptive statistics of Table 1, the total innovations were on average 0.9 in manufacturing firms and 0.8 in firms in the services sectors, which indicates a low value in the innovation performance.

**Table 1:** Descriptive statistics manufacturing industry.

Variable	Obs	Mean	Std. Dev.	Min	Max
Total Innovations	6798	.927	3.792	0	189
Process Innovations	6798	.227	.982	0	25
Product innovations	6798	.485	2.927	0	185
Marketing Innovations	6798	.117	.474	0	10
Organizational Innovations	6798	.098	.495	0	10
Size (logs)	6798	3.731	1.305	.405	8.01
R&D Intensity (logs)	654	5.738	2.06	-3.252	10.474
Qualified Personnel	6798	.316	.222	0	1
High Qualified Personnel	6798	.005	.017	0	.5
Demand Pull	6798	.216	.411	0	1
Open Innovations	6798	.317	.832	0	9
Confidentiality agreements	6798	.132	.339	0	1
Open Innovations and Confidentiality agreements	6798	.06	.238	0	1

Source: EDIT-EDITS-DANE.

**Table 2:** Descriptive statistics services sector.

Variable	Obs	Mean	Std. Dev.	Min	Max
Total Innovations	9304	.83	6.273	0	436
Product Innovations	9304	.475	5.967	0	436
Process innovations	9304	.105	.593	0	23
Marketing Innovations	9304	.101	.419	0	11
Organizational Innovations	9304	.149	.648	0	32
Size (logs)	9304	4.384	1.295	.405	9.296
R&D Intensity (logs)	868	6.095	2.017	-.157	12.678
Qualified Personnel	9304	.422	.283	0	1
High Qualified Personnel	9304	.016	.062	0	1
Demand Pull	9304	.242	.428	0	1
Open Innovations	9304	.384	1.022	0	9
Confidentiality agreements	9304	.163	.369	0	1
Open Innovations and Confidentiality agreements	9304	.068	.252	0	1

Source: EDIT-EDITS-DANE.

The results in Tables 3 and 4 suggest that open innovation is positively associated with overall innovative performance in the manufacturing and service industries. Companies that reported using open innovation also reported higher levels of innovative performance, such as a higher number of patents filed, a higher number of new product introductions, and a higher percentage of revenue from new products. However, the results also suggest that the use of confidentiality agreements is a critical factor in maximizing the benefits of open innovation.

**Table 3:** Zero inflated poisson models manufacturing industry.

	(1)	(2)	(3)	(4)	(5)	(6)	(7)	(8)	(9)	(10)
VARIABLES	Total Innovation	inflate	Product Innovations	inflate	Process innovation	inflate	Marketing Innovations	inflate	Organizational Innovation	inflate
Size(logs)	0.247***	0.157	0.302***	0.428***	0.205***	-0.133	0.203***	0.129	0.231***	0.0595
	(0.0133)	(0.290)	(0.0179)	(0.111)	(0.0356)	(0.105)	(0.0610)	(0.181)	(0.0613)	(0.137)
R&D Intensity (logs)	0.132***	0.329*	0.138***	-0.0170	0.139***	0.0396	-0.0650*	-0.221**	0.0458	-0.0555
	(0.0100)	(0.196)	(0.0141)	(0.0693)	(0.0289)	(0.0908)	(0.0385)	(0.0887)	(0.0434)	(0.0886)
Qualified Personnel	-0.124	1.186	0.0453	0.314	-0.0971	1.637*	1.712***	4.646***	0.950*	2.502**
	(0.109)	(2.171)	(0.147)	(0.843)	(0.321)	(0.861)	(0.469)	(1.387)	(0.494)	(1.042)
High Qualified Personnel	3.064***	-65.73	1.623	1.719	5.092***	0.0320	5.025	9.624	-2.358	-12.53
	(0.786)	(51.36)	(1.253)	(7.873)	(1.734)	(5.663)	(3.733)	(7.627)	(3.417)	(9.637)
Demand Pull	1.242***	-17.79	1.862***	-2.466***	0.301	-1.657***	1.190***	0.691	1.356***	0.760
	(0.130)	(488.7)	(0.523)	(0.705)	(0.210)	(0.376)	(0.448)	(1.392)	(0.516)	(1.490)
Open Innovation (dummy)	0.325***	1.455	0.101	-0.704**	0.110	-0.465	0.0138	-0.691	0.0754	-0.394
	(0.0598)	(1.042)	(0.0859)	(0.343)	(0.157)	(0.418)	(0.251)	(0.505)	(0.346)	(0.573)
Confidentiality agreements	-0.157	0.687	-0.183	0.417	-0.207	-0.324	-1.107***	-14.85	-0.775**	-13.19
	(0.100)	(1.374)	(0.154)	(0.452)	(0.257)	(0.637)	(0.336)	(750.6)	(0.372)	(418.2)
Open Innovation X Confidentiality agreements	0.543***	-1.535	0.580***	-0.852	0.529*	0.503	1.325***	14.80	1.224***	13.07
	(0.108)	(1.581)	(0.164)	(0.550)	(0.275)	(0.713)	(0.383)	(750.6)	(0.408)	(418.2)
Constant	-1.990***	-4.090*	-3.197***	-0.974	-1.859***	0.766	-2.715***	-2.037	-3.321***	-1.229
	(0.166)	(2.219)	(0.534)	(1.061)	(0.345)	(0.859)	(0.579)	(1.606)	(0.728)	(2.000)
Observations	654	654	654	654	654	654	654	654	654	654

Standard errors in parentheses.

*** p<0.01

** p<0.05

* p<0.1.Source: EDIT-EDITS-DANE.

**Table 4:** Zero inflated poisson models services sector.

	(1)	(2)	(3)	(4)	(5)	(6)	(7)	(8)	(9)	(10)
VARIABLES	Total Innovation	inflate	Product Innovations	inflate	Process innovation	inflate	Marketing Innovations	inflate	Organizational Innovation	inflate
Size(logs)	0.396***	0.138	0.448***	0.162*	0.359***	0.00411	0.275***	0.154	0.305***	0.154
	(0.0112)	(0.149)	(0.0145)	(0.0889)	(0.0402)	(0.0871)	(0.0482)	(0.131)	(0.0413)	(0.151)
R&D Intensity (logs)	0.175***	0.309***	0.201***	0.133**	0.153***	0.0198	0.176***	0.290**	0.118***	0.226
	(0.00859)	(0.120)	(0.0111)	(0.0608)	(0.0273)	(0.0605)	(0.0499)	(0.143)	(0.0362)	(0.142)
Qualified Personnel	0.750***	0.811	0.652***	-2.717***	0.404	0.436	-0.290	-1.005	0.179	0.142
	(0.103)	(1.044)	(0.159)	(0.562)	(0.311)	(0.636)	(0.459)	(1.379)	(0.332)	(1.230)
High Qualified Personnel	3.053***	-0.767	3.641***	-7.759***	-0.168	1.419	-0.267	1.658	-0.109	-0.898
	(0.108)	(1.599)	(0.155)	(1.305)	(0.439)	(0.876)	(0.780)	(1.639)	(0.391)	(1.450)
Demand Pull	1.151***	-3.097***	0.833***	-3.647***	2.710***	14.01	0.516	-1.359	1.127***	0.0951
	(0.150)	(0.452)	(0.289)	(0.504)	(0.313)	(709.1)	(0.475)	(0.833)	(0.384)	(1.241)
Open Innovation (dummy)	0.382***	0.102	0.185	-0.910***	0.0774	-0.379	0.469	0.421	0.0109	-1.210*
	(0.0715)	(0.610)	(0.123)	(0.341)	(0.190)	(0.333)	(0.298)	(0.952)	(0.229)	(0.629)
Confidentiality agreements	0.828***	0.361	0.966***	-0.542	-0.106	-0.492	1.124***	1.237	0.211	-0.665
	(0.0819)	(0.707)	(0.132)	(0.384)	(0.266)	(0.486)	(0.312)	(0.912)	(0.270)	(0.670)
Open Innovation X Confidentiality agreements	-0.449***	-0.868	-0.473***	0.254	0.0935	0.170	-1.057***	-1.322	0.0777	0.574
	(0.0887)	(0.834)	(0.139)	(0.475)	(0.288)	(0.541)	(0.357)	(1.019)	(0.289)	(0.791)
Constant	-4.256***	-3.423**	-4.762***	3.387***	-5.722***	-14.04	-3.945***	-2.258	-3.812***	-2.536*
	(0.184)	(1.562)	(0.345)	(0.901)	(0.434)	(709.1)	(0.623)	(1.526)	(0.455)	(1.464)
Observations	868	868	868	868	868	868	868	868	868	868

Standard errors in parentheses.

*** p<0.01

** p<0.05

* p<0.1.Source: EDIT-EDITS-DANE.

The results of the zero-inflated Poisson regression models indicate in the manufacturing industry that confidentiality agreements generate negative effects on marketing and organizational innovations and in the services sector a positive result of confidentiality agreements on total innovations and innovations. products. This result shows an interesting contrast in the economic sectors, in favor of the regularization and risk mitigation of trade secrets and intellectual property of a company in the service sector and manifesting a barrier at the time of generating good innovative performance in an industrial company.

The findings also suggest that confidentiality agreements are essential to protect a company’s trade secrets and intellectual property in the service sector. Companies reported that they use confidentiality agreements to manage the risks associated with open innovation and that they see these agreements as an important tool to protect their intellectual property. The study also identified best practices for managing confidentiality in open innovation.

Tables 3 and 4 show us interesting results to analyze in the control and interest variables of this research. The Schumpeterian hypothesis related to company size is fulfilled. This tells us that as the company, regardless of the sector to which it belongs, is larger, it will support greater technological and non-technological innovative performance. Larger companies usually have the greater resources to hire highly qualified workers specialized in the development of innovations, have greater financial capacity to support R&D activities, and easily take advantage of economies of scale for production and relations with the environment. All these aspects result in greater innovative performance.

Except for marketing innovations in the manufacturing industry, there is a positive and significant effect of R&D intensity on technological and non-technological innovative performance for both sectors. A greater financial resource in R&D implies that companies have released resources available to support the activities and the effort to carry out an innovation that could result in the development of novelties or improvements in products, processes, marketing, and organizational techniques to strengthen productivity and competitiveness of companies.

An interesting result is found in the economic sectors in terms of qualified personnel. There is a positive effect on fostering organizational and marketing innovations in the manufacturing industry and a positive effect on product and process innovations in the service sector. Likewise, a positive effect of highly qualified personnel is generated on the total innovative performance of both sectors. The positive result on innovative performance implies the use of better information, greater knowledge, and problem-solving skills regarding the development of innovations, better experience, and opportunities for training and development of products, processes, and techniques. Qualified personnel also absorb knowledge much faster, which they translate into technological and non-technological innovations.

The importance that the company and consumers give to the improvement in the quality of the goods or services and the expansion in the range of goods or services offered [26] (Griffith et al., 2006), is reflected in better innovative performance for both sectors. Behind this importance are factors associated with the improvement of goods and services as this importance is supported by a greater reflection of productivity due to idiosyncratic demand shocks and greater collaboration for the efficient development of innovations within companies.

There is evidence of positive effects of confidentiality agreements on types of innovations (products, processes, and marketing) in the service sector and negative effects on some types of the manufacturing industry (marketing and organization) and there is evidence of a positive effect of open innovation on total innovative performance. Likewise, the results show that the interaction of open innovation and confidentiality agreements generates positive effects on the innovative performance of the manufacturing industry and negative ones in the services sector.

Open innovation practices allow companies to access a broader range of knowledge and experience, leading to more innovative ideas and technologies [27,28]. Meanwhile, nondisclosure agreements allow companies to protect their intellectual property and sensitive information, reducing the risk of competitors stealing their ideas and making it easy to innovate. However, non-compete clauses or other restrictive agreements may be generated in confidentiality agreements, which prevent the flow of talent and ideas between companies and limit innovation.

The positive results in the interaction support the concept of legitimate trust and collaboration of the manufacturing companies. Well, once confidentiality agreements are generated, open innovation relationships encourage technological and non-technological innovations. While the negative effect on the services sector supports caution in information and knowledge. This is because service companies tend to be more cautious when it comes to sharing information and knowledge with other market players. In addition, innovation in the service sector usually involves customization and adaptation to the specific needs of each client, which makes collaboration with other companies difficult.

## Discussion

The results of this study suggest that open innovation and confidentiality agreements are key factors in driving innovative performance in the manufacturing and service industries. However, the collaborative nature of open innovation can create challenges around confidentiality and intellectual property. The results suggest that the use of confidentiality agreements is a critical factor in maximizing the benefits of open innovation. Companies must find a balance between collaborating with external partners and protecting their confidential information and trade secrets to fully realize the benefits of open innovation.

In short, while non-disclosure agreements can provide benefits to companies in terms of protecting their industrial and intellectual property, they can also have negative effects on innovation by limiting collaboration, information sharing, and the flow of talent and ideas. Companies must balance the potential benefits and risks associated with confidentiality agreements and consider alternative approaches to protect their intellectual property, such as patent protection or open innovation models.

The paper discusses the implications of the findings for companies in the manufacturing and service industries. The results suggest that the interaction of open innovation and confidentiality agreements generates positive effects on the innovative performance of the manufacturing industry and negative effects on the service sector. This suggests that the positive effect of open innovation and confidentiality agreements in the manufacturing industry is due to its greater tradition of collaborative innovation, while in the service sector, companies tend to be more cautious when it comes to sharing information and acquaintance with other market players. Therefore, companies should adopt open innovation practices to improve their innovative performance, while using confidentiality agreements to protect their industrial and intellectual property and confidential business information. However, the paper notes that there are potential drawbacks to using confidentiality agreements, such as the risk of limiting collaboration with external partners.

### Conclusions

The interaction of open innovation and confidentiality agreements generates positive effects on the innovative performance of the manufacturing industry and negative effects on the services sector. This suggests that the positive effect of open innovation and confidentiality agreements in the manufacturing industry is due to its greater tradition of collaborative innovation, while in the service sector, companies tend to be more cautious when it comes to sharing information and knowledge of other market players.

The positive effect of open innovation and confidentiality agreements in the manufacturing industry stems from its long tradition of collaborative innovation. In manufacturing, nondisclosure agreements are used to protect ideas and products in development, while enabling collaboration and knowledge sharing with other companies. In the services sector, confidentiality agreements can harm open innovation because service companies are often more cautious when sharing information and knowledge with other market players. In addition, innovation in the service sector is often based on customization and adaptation to the specific needs of each client, which makes collaboration with other companies difficult.

In summary, the effect of open innovation and confidentiality agreements depends on the industry context in which they are used. In the manufacturing industry, these mechanisms can have a positive effect on innovation, while in the service sector, they can harm open innovation.

## References

[1] Chesbrough H. Open innovation: The new imperative for creating and profiting from technology. Harvard Business Press; 2003.

[2] Zhang X, Chu Z, Ren L, Xing J. Open innovation and sustainable competitive advantage: The role of organizational learning. Technological Forecasting and Social Change. 2023;186. doi: 10.1016/j.techfore.2022.122114

[3] Alves JL, de Carvalho MM. Knowledge management and project uncertainty in open innovation context: Trends and contributions of literature. Brazilian Journal of Operations and Production Management. 2023;20(1). doi: 10.14488/BJOPM.1530.2023

[4] Gassmann O, Enkel E. Towards a Theory of Open Innovation: Three Core Process Archetypes. University of St.Gallen; 2004.

[5] De Andrés-Sánchez J, Musiello-Neto F, Rua OL, Arias-Oliva M. Configurational Analysis of Inbound and Outbound Innovation Impact on Competitive Advantage in the SMEs of the Portuguese Hospitality Sector. Journal of Open Innovation: Technology, Market, and Complexity. 2022;8(4):205. doi: 10.3390/joitmc8040205

[6] Chesbrough H, Brunswicker S. A Fad or a Phenomenon?: The Adoption of Open Innovation Practices in Large Firms. Research-Technology Management. 2014;57(2):16–25. doi: 10.5437/08956308X5702196

[7] Dahlander L, Gann DM. How open is innovation?. Research policy. 2010;39(6):699–709.

[8] Chinchilla Imbett CA. Propiedad privada y derechos adquiridos en el proceso de formalización y clarificación de la propiedad del Decreto 902 de 2017 a la luz de los principios generales del derecho: la buena fe y la confianza legítima. Revista Derecho del Estado. 2018;(41):147–171. doi: 10.18601/01229893.n41.06

[9] Chesbrough H, Lettl C, Ritter T. Value creation and value capture in open innovation. Journal of Product Innovation Management. 2018;35(6):930–938. doi: 10.1111/jpim.12471

[10] Chen X, Zhang H, Wang B. How formal and informal open innovation impact innovation performance: The moderating role of appropriability. Technology Analysis and Strategic Management. 2022. doi: 10.1080/09537325.2022.2153661

[11] Nguyen TPT, Huang F, Tian X. Intellectual property protection need as a driver for open innovation: Empirical evidence from vietnam. Technovation. 2023;123. doi: 10.1016/j.technovation.2023.102714

[12] Luoma T, Paasi J, Valkokari K. Intellectual property in inter-organisational relationships—findings from an interview study. International Journal of Innovation Management. 2010;14(3):399–414. doi: 10.1142/S1363919610002702

[13] Ford SJ, Mortara L, Probert DR. Disentangling the complexity of early-stage technology acquisitions. Research Technology Management. 2012;55(3):40–48. doi: 10.5437/08956308X5503048

[14] Lu SC, Kong DT, Ferrin DL, Dirks KT. What are the determinants of interpersonal trust in dyadic negotiations? Meta-analytic evidence and implications for future research. Journal of Trust Research. 2017;7(1):22–50. doi: 10.1080/21515581.2017.1285241

[15] Grimaldi M, Greco M, Cricelli L. A framework of intellectual property protection strategies and open innovation. Journal of Business Research. 2021;123:156–164. doi: 10.1016/j.jbusres.2020.09.043

[16] Rosell DT, Lakemond N, Melander L. Integrating supplier knowledge in new product development projects: decoupled and coupled approaches. Journal of Knowledge Management. 2017;21(5):1035–1052. doi: 10.1108/jkm-10-2016-0438

[17] Bryan S. Confidentiality agreements: Necessary, but not always sufficient. Journal of Business Strategy. 2015;36(5):27–33.

[18] Wadhwa M, Harper A, eds. Technology, Innovation, and Enterprise Transformation. IGI Global; 2015. doi: 10.4018/978-1-4666-6473-9

[19] Segura L del P. Alcances de la confianza legítima en el derecho privado colombiano. Cuadernos De La Maestría En Derecho. 2015;(3). https://revistas.usergioarboleda.edu.co/index.php/Cuadernos/article/view/428.

[20] Gorbatyuk A, Van Overwalle G, van Zimmeren E. Intellectual Property Ownership in Coupled Open Innovation Processes. IIC. 2016;47:262–302. https://doi-org.proxy.bidig.areandina.edu.co/ doi: 10.1007/s40319-016-0461-1

[21] Hallberg NL, Brattström A. Concealing or revealing? Alternative paths to profiting from innovation. European Management Journal. 2019;37(2):165–174. doi: 10.1016/j.emj.2018.04.003

[22] Noh H, Lee S. What constitutes a promising technology in the era of open innovation? An investigation of patent potential from multiple perspectives. Technological Forecasting and Social Change. 2020;157:120046. doi: 10.1016/j.techfore.2020.120046

[23] Toma A, Secundo G, Passiante G. Open innovation and intellectual property strategies. Business Process Management Journal. 2018;24(2):501–516. doi: 10.1108/bpmj-11-2016-0230

[24] Bigliardi B, Galati F. Models of adoption of open innovation within the food industry. Trends in Food Science & Technology. 2013;30(1):16–26. doi: 10.1016/j.tifs.2012.11.001

[25] Melgar MC, Ordaz JA, Guerrero FM. Diverses alternatives pour déterminer les facteurs significatifs de la fréquence d’accidents dans l’assurance automobile. Insurance & Risk Management. 2005;73(1):31–54.

[26] Griffith R, Huergo E, Mairesse J, Peters B. Innovation and productivity across four European countries. Oxford Review of Economic Policy. 2006;22(4):483–498.

[27] Cohen WM, Levinthal DA. Absorptive capacity: A new perspective on learning and innovation. Administrative science quarterly. 1990;35(1):128–152.

[28] Cui Y, Jiang Y. Open innovation in the manufacturing industry: A case study. Journal of technology management in China. 2012;7(2):204–219.

